# Drivers and barriers to acceptance of human-papillomavirus vaccination among young women: a qualitative and quantitative study

**DOI:** 10.1186/1471-2458-10-68

**Published:** 2010-02-14

**Authors:** Gitte Lee Mortensen

**Affiliations:** 1 AnthroConsult, Fynsgade 24, 2th, 8000 Aarhus C, Denmark

## Abstract

**Background:**

Human papillomavirus (HPV) is a necessary cause of cervical dysplasia and cancer, and of genital warts. Few studies have examined attitudes to HPV vaccination since the introduction of HPV vaccines. We aimed to investigate the reasons for young women's acceptance or rejection of the quadrivalent HPV vaccine after its general availability in Denmark.

**Method:**

A literature review assessed attitudes towards HPV vaccination and the information was used to identify relevant questions for telephone and focus group interviews with women aged 16-26 who had decided to receive or reject HPV vaccination. 435 women across Denmark were interviewed by telephone. Qualitative interviews were undertaken in focus groups with 33 women living in Odense who had completed the telephone survey. Four focus groups were set up according to age (16-20 and 21-26 years of age) and acceptance/rejection of the vaccine.

**Results:**

Of 839 women initially contacted by telephone, 794 were included, 411 (49%) said they accepted vaccination but only 201 (24%) had actually received the vaccine and these latter were interviewed. 242 women said they refused vaccination of which 234 were interviewed. Women who were undecided were excluded from the study. Prevention of cervical cancer was the main driver for acceptance of the vaccine, followed by parental encouragement and financial support, personal experience of someone with cancer and recommendation by health-care professionals. The greatest barrier to vaccination was its cost. A lack of information about the benefits of vaccination for sexually active women was also an important barrier and the older participants in particular considered that they were too old to be vaccinated. Knowledge about HPV and its role in the development of cervical cancer and genital warts was poor.

**Conclusions:**

The difference between intention to be vaccinated and starting vaccination was considerable, and a large proportion of women aged 16-26 did not wish to be vaccinated. If the most important barriers to vaccination were addressed (cost and a lack of information about vaccination benefits), it is likely that the uptake of vaccination in Denmark would increase substantially.

## Background

Persistent infection with certain sexually transmitted types of human papillomavirus (HPV) is a necessary cause of cervical dysplasia and cancer, and of genital warts [1]. In Europe, it is estimated that 37,500 women are affected by cervical cancer every year [2]. Genital warts are one of the most prevalent sexually transmitted diseases in Europe; the cumulative incidence in women in four Nordic countries has been estimated at more than 10% [3].

Two vaccines against HPV have recently become available - the bivalent vaccine Cervarix^® ^(GSK) and the quadrivalent vaccine Gardasil^® ^(Merck) - both of which protect against HPV types 16 and 18, which cause about 70% of cervical cancers. The quadrivalent vaccine also protects against infection with HPV types 6 and 11, which cause about 90% of cases of genital warts [4-9]. HPV vaccine coverage is thus an important public health concern and understanding the reasons for accepting or rejecting vaccination is essential for increasing compliance.

### Attitudes before introduction of the vaccine

A number of studies have examined the potential reception of a HPV vaccine among parents and young people before approval and widespread introduction. A systematic review of international literature published before January 2007, that was carried out in connection with a Danish Health Technology Assessment (HTA) of HPV vaccination [10], showed that attitudes towards HPV vaccination were mostly positive and that 70-90% of parents said they wanted to have their children vaccinated [10-18]. The intention to vaccinate depended on a number of factors, including the knowledge that HPV is the cause of cervical cancer, confidence in the vaccine's safety and efficacy [11,12,14,16-22], and the perception of HPV infection or cervical lesions as common occurrences with a potentially serious outcome [12,15,17,21-24]. Parents were also influenced by the cost of the vaccine [12,18,21,24,25] and whether it had been recommended by a general practitioner (GP) or 'significant others' (e.g. family member) [12,13,17,18,21,22]. Some USA-based studies found that parents' fear that HPV vaccination might be seen as an endorsement of promiscuity was a barrier to acceptance [11,13,16,19,21,22]. Parental acceptance of vaccination also depended on the age [11,16,19,20,22] and sex [15,20,22] of their children, as well as their own experiences of sexually transmitted infections (STI) or cancer [21,24].

The empirical studies of the Danish HTA of HPV vaccination showed that the majority of parents and young men and women interviewed had positive attitudes towards HPV vaccination [10], although the participants expressed a need for more information about the vaccine and its safety. Most felt that HPV vaccination should be integrated into the free childhood-immunisation programme to secure equal access and to ensure that vaccination was achieved before sexual debut. However, parents of teenagers also wanted their children vaccinated, despite the possibility that they had already been exposed to HPV. Participants aged 18-22 years expressed a desire to be vaccinated, but doubted they would actually do so if they had to pay for it themselves. The knowledge that HPV is sexually transmitted did not have a negative effect on Danish participants' attitudes towards the vaccine; vaccination was not seen as stigmatising or promoting sexual promiscuity and men and women were considered to be equally responsible for the prevention of sexually transmitted infections (STIs).

### Attitudes after introduction of the vaccine

The participants in the previously mentioned studies were asked about their attitudes to the HPV vaccine *before *it became readily available or available free-of-charge. As an expression of intentions regarding a hypothetical situation, such results may be an imprecise indicator of behaviour in a real situation [26]. The literature review we carried out in preparation for the present study showed that few studies have been carried out on attitudes to and acceptance of HPV vaccination *after *the vaccine has been approved and has become readily available in many countries.

Since the approval of the first HPV vaccine in 2006 in the USA [27], many countries have introduced HPV vaccination into national health-care systems, although the choice of the vaccine recommended, organisation and financing, and the age and sex of the recipients vary considerably. Most countries have selected the quadrivalent vaccine and while most recommend HPV vaccination for girls only, Austria and Greenland also offer the vaccine to boys [9].

In Denmark, the quadrivalent vaccine was approved and became available in September 2006. Since January 2009 it has been offered free-of-charge within the childhood-immunisation programme for girls aged 12 years. A catch-up programme for girls aged 13-15 years (girls born in 1993-95) started in October 2008. The Danish National Board of Health informs all girls in this target group directly by mail and has produced information material about HPV vaccination and cervical cancer which is available in doctors' offices and on the Internet. The Danish Cancer Society is conducting an information campaign that has included the training of 200 'ambassadors', i.e. young women who give lectures on HPV vaccination at educational institutions across the country. The vaccine manufacturer has also run a media-based information campaign focused on 'vaccination against cervical cancer'.

The uptake of free HPV vaccination in Denmark has been massive, and by January 31st 2009, 71% of girls born in 1993, 76% of girls born in 1994, and 67% of girls born in 1995 had been vaccinated [28]. While the vaccine is also approved for women aged 16-26 years, these women, like all boys and men, must pay about 470 euros if they want to be vaccinated.

The aim of the present study, the first of its kind in a Danish context, was to examine the attitudes to HPV vaccination of young women aged 16-26 years after the quadrivalent HPV vaccine became readily available and after its introduction into the free childhood-immunisation programme. Now that childhood HPV vaccination is actually available, are young Danish women above the age of 15 taking the initiative to be vaccinated and what are the factors determining acceptance or refusal of vaccination?

## Methods

For this study, we used methodological triangulation to increase the reliability and validity of the results [29]. A telephone interview-based survey based on a structured multiple-choice questionnaire and qualitative focus group interviews centred on issues identified in the previously described literature review. The quantitative part of the study was used to investigate patterns and frequency of attitudes towards vaccination. The qualitative and central approach involving focus group interviews was used to examine in depth perceptions of the diseases covered by HPV vaccination, treatments and preventive methods, enabling us to explain patterns of meaning related to attitudes towards HPV vaccination. We thus acquired knowledge about the qualities of the phenomenon regardless of its frequency [30]. The qualitative research methods also allowed us to explore the reasons for differences between intention and action in this particular socio-cultural context [31], and were particularly suitable for examining health-related choices concerning the prevention of HPV infection. The study did not require approval from the Danish National Committee on Biomedical Research Ethics. The methodology and analyses were discussed in an ad-hoc committee formed by the author, the assistant anthropologist, an independent, experienced anthropologist and a statistician.

### Literature review

To supplement the literature review carried out by the author in connection with the Danish HTA [10], we performed an updated literature search on PubMed using the English search terms (MeSH and free text; singular and plural terms): <HPV>, <human papilloma virus>, <wart-virus>, <vaccines>, <vaccination>, <immunization>, <vaccine programme> combined with <parents>, <youth>, <young>, <youngster>, <people>, <adolescent>, <teenage>, <attitude>, <acceptance>, <accept>, <knowledge>, <barriers>, <cultural> to identify papers published between January 2007 and January 2009. Danish search terms: <Vaccination> (vaccination), <patient> (patient) combined with <holdninger> (attitudes), <accept> (acceptance) and <viden> (knowledge) were also used. The papers found were used to identify relevant questions for the participants in the telephone interviews and focus groups.

### Telephone interviews

Telephone interviews were chosen as a method of presenting a statistically significant number of women with a limited range of open-ended questions regarding attitudes towards HPV vaccination. A structured-question interview guide and computer-assisted telephone interviewing (CATI) software (Sawtooth) was used by professional interviewers (DMA/Research, Aarhus, Denmark). The guide included questions on the level of education of the women and their parents, the sources of their knowledge about HPV, if and with whom they had discussed HPV vaccination, and the main reasons for their choice. Based on the literature review, a number of predefined response options to each question were inserted in the CATI guide for the interviewers to mark if the participant's response corresponded to it, for instance "because my parents paid for it" or "because I've already been sexually active". Multiple responses were allowed. For each question, the CATI guide also included an open option ('other') that allowed the interviewers to fill in answers not corresponding to the predefined response options using free text. These free text answers were subsequently coded into main topics. In some cases, these answers could be included in the predefined response options after all; in other cases new response categories were created.

**Figure 1:**
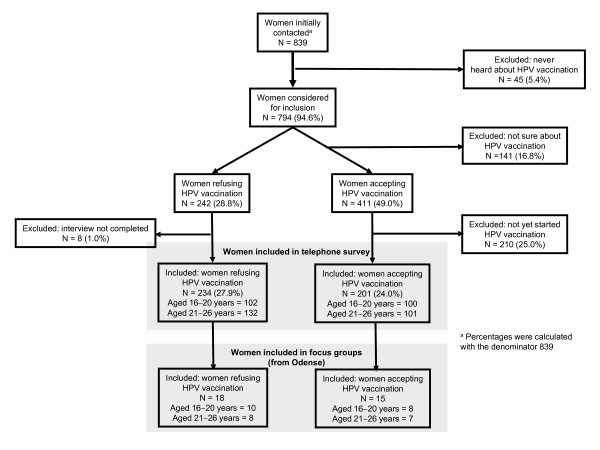
**Description of groups of participants in the telephone survey and focus groups**.

For the telephone interviews, participants were recruited via randomised calls to a geographically representative sample of households in Denmark in mid January 2009. To avoid a regional or city/country bias, this method implied calling publicly available telephone numbers to households across the country without prior knowledge about their residents. Women aged between 16-26 years who had heard of HPV vaccination were informed about the purpose of the study and invited to participate anonymously. To be included in the 'accepting HPV vaccination' group, they had to have already started or finished vaccination. Those who had decided not be vaccinated were included in the 'refusing HPV vaccination' group. Those who were undecided were excluded from the study (figure 1). Telephone interviews were ended when a representative number of women, corresponding to each region's quota of the total population, had participated.

### Focus group interviews

Focus group interviews were chosen with the aim of creating a confidential setting where women in a comparable situation could openly discuss their reflections and choices. The volunteers were divided into four groups by acceptance or rejection of HPV vaccination and by age to encourage more open and honest discussion [32]. The aim was to gain an insight into as broad a range of perspectives on HPV vaccination as possible. As a social research method, the combination of group interaction and the focus on a particular topic in focus groups are suitable for producing empirical data on the social construction of meaning. This includes those social repertoires of meaning that are usually taken for granted and that people draw on, interpret and act on in their everyday practices along with significant others [32,33]. One of the purposes of a focus group is to help the participants express relevant parts of this silent knowledge [34]. Focus groups can thus generate an insight into the norms at base of certain practices and of the level of consensus and conflicts in a given area [35,36].

Focus group interviews were carried out with women who had completed the telephone interview and who were living in Odense. Participants were invited immediately following the telephone interview on a first come, first served basis until a sufficient number of participants for each group was attained. Volunteers were divided into four groups: two groups of women who accepted HPV vaccination and had begun or completed the vaccination series (eight women aged 16-20 years and seven women aged 21-26 years) and two groups who did not want to be vaccinated (ten women aged 16-20 years and eight women aged 21-26 years). No additional personal information was collected about these women, who gave their informed consent and also participated anonymously in the focus groups. Participants also only heard the first names of each other. A total of 33 women, all ethnic Danes and mostly students, participated in the focus group discussions (Figure 1).

The focus groups were moderated by the author and a colleague, using a semi-and funnel structured interview guide that involved going from general questions about attitudes to vaccines in general to more specific questions about HPV vaccination. Open-ended questions were used to capture information not anticipated by the literature review [32]. The discussions were transcribed verbatim and analysed using specific software for handling qualitative data, Nvivo (QSR International). A discourse theoretical approach to the relations between language and the social construction of meaning was used to analyse the data [37]. This comprised an analysis of the terminology used to speak about the subject and the ways this related to other issues. First, the data were coded by topic raised during the discussions and then the most important themes within each topic were identified. The frequency and connections between topics and themes was then analysed to obtain a pattern of the relative importance of the different topics and themes for the participants and thus the significant drivers for and barriers against HPV vaccination. All methodological and analytical steps were discussed and alternative interpretations sought with the assisting anthropologist, an additional anthropologist not involved in the project and a statistician. Disagreements concerning the focus groups were solved using Spradley's analytical process of resolution of qualitative data [38]. The quotes given in the results section illustrate the participants' most pertinent reflections.

## Results

### Results of the updated literature review

The results from the updated literature review mostly showed support for the results of earlier studies [39-49]. Among the studies conducted before widespread availability of the vaccine, some suggested that the perception of whether the vaccine is aimed at preventing HPV infection, cervical cancer or genital warts can be decisive to acceptance [48,49]. Jones & Cook showed that among male university students in the USA, interest in HPV vaccination rose considerably when the vaccine was presented as a means of preventing genital warts [46]. They also suggested that personal experiences (e.g. number of sexual partners, history of STIs or HPV infection) more often acted as drivers rather than barriers to acceptance of HPV vaccination [46].

Only a few of the identified studies had been conducted after the introduction of HPV vaccination [50-53]. Stretch et al. described how the attitudes of parents in the UK had been confirmed rather than altered by receiving more information about HPV and cervical cancer [53]. Ogilvie et al. reported that 70% of Canadian parents said they would like their daughters to benefit from free vaccination against HPV, although it was not reported how many girls had actually started the vaccination series [51]. In a USA-based study by Rosenthal et al., 26% of health-care providers had started or completed the vaccination of adolescent girls in their care [50]. Also, n the USA, Kahn et al. reported that while 66% of the young women in their study intended to be vaccinated against HPV, only 5% had actually started [52]. These few studies indicate that, although intention to vaccinate remains strong when the HPV vaccine becomes available in a country, there can be considerable discrepancy between intention and action.

### Survey sample

Of the 839 women initially contacted by telephone, 794 (94.6%) had heard of HPV vaccination and was thus considered for inclusion (Figure 1). 141 women (corresponding to 16.8% of all women contacted) were excluded from participation in the study, however, because they were undecided as to whether they wanted HPV vaccination. 242 women (corresponding to 28.8% of all women contacted) stated that they did not want to be HPV vaccinated. Among these women, 234 telephone interviews were completed. 411 women (49% or nearly half of all women contacted) stated that they wanted HPV vaccination, but only approximately half of these women, i.e. 201 women (24%) had actually started or completed the vaccination series and were included for telephone interviewing. 210 of the women stating to want HPV vaccination (25% of all women contacted) were thus excluded. A total of 435 women in the relevant age group were interviewed by telephone. The majority of women in the survey had low income, most being students (81.5%); although tuition in Denmark is free, the average grant for students living alone is €683. The others were working (14.2%), apprentices (2.1%) and unemployed (2.2%).

### Focus groups: Perceptions and knowledge about HPV vaccination and HPV-related diseases

Overall, the focus group participants' views on vaccines already integrated into the Danish childhood-immunisation programme and travel vaccines were positive. The participants said that vaccination was a good health-care intervention which is routinely accepted. Nor were the women who refused HPV vaccination against HPV vaccination as such.

The focus group participants' knowledge about HPV as an infectious agent, and the prevalence of cervical dysplasia and cervical cancer was poor, for both those who accepted and those who refused HPV vaccination. With a few exceptions, the women were not aware that the vaccine only protects against cervical cancers that are caused by HPV 16 and 18 (representing about 70% of all cancers); most thought that all cervical cancers could be prevented if vaccination occurred before sexual debut. This misconception was partly founded on perceptions of the nature of vaccines in general, and was related to the idea that the vaccine would not have been included in the Danish childhood-immunisation programme if this was not the case. The focus groups participants were uncertain of the benefits of vaccination for themselves because most of them were already sexually active. Their knowledge about genital warts was also poor, particularly among those who refused HPV vaccination; only a few women in the focus group of 21-26-year-olds who accepted HPV vaccination knew that the vaccine can also prevent genital warts.

None of the focus group participants had received information about HPV vaccination from their GP, and only one had received information from her gynaecologist. Most of them had heard about vaccination against cervical cancer from the media-information campaign. However, many focus group participants stated that they did not feel concerned because the campaign seemed to target girls aged 12-15 years, and the women refusing HPV vaccination tended to feel under-informed and outside the target group for vaccination.

There simply hasn't been enough information clearly saying: "hey, this one's intended for you too!" - or for my age group. I had no idea what the criteria were. That's not given anywhere. I haven't heard about that at all. In this situation, I just think that as I hadn't found out about it either in school or in the news, how important can it be, when there is so little information? [18-year-old focus group participant refusing HPV vaccination]

Many of the focus group participants refusing vaccination felt that the National Board of Health should contact young women over 15 years old (as they do for HPV vaccination of 12-15 year old girls and other health-care issues), and inform them directly about the efficacy of the vaccine in sexually active women [5].

Once - I don't remember if it was when I turned 18 - I received a letter [from the National Board of Health] inviting me to go for a check-up for chlamydia. I don't understand why they don't do that with this as well. They could send a letter to those who don't get the vaccine for free, inform them about HPV and cervical cancer and other diseases and about the availability of a vaccine, and advise them to go to see their GP for more information, or something like that, so that we'd learn a bit more about it. Because it seems that a lot of us haven't heard of this before and that's quite worrying when you think about how serious it is. They should do more to reach out to those who are exposed. [20-year-old focus group participant refusing HPV vaccination]

The focus groups showed that the sexual transmission of HPV was associated with a perception of a high risk of infection because sex was regarded as a natural part of life and not something one could (or would want to) avoid. This was in contrast to smoking or sun-bed use, for example, that were often cited as behaviours associated with an increased risk of cancer. Thus, for the focus group participants, the fact that HPV vaccination protects against a sexually transmitted disease was not a barrier to the acceptance of vaccination.

### Results across the quantitative and qualitative studies: Drivers for HPV vaccination

#### Prevention of cervical cancer

The results from the survey showed that prevention of cervical cancer was the main reason for accepting HPV vaccination, with parental advice and experience of cancer with close relatives as the second and third most frequent reasons (Table 1). In the focus groups with women accepting HPV vaccination, the women elaborated on this by saying that any measure to prevent cancer was welcome. For some, the acceptance of vaccination was higher because they knew somebody who had had cancer. Several focus group participants said that they thought cancer and related diseases were frequent and that they were as likely as anyone to become ill.

**Table 1:** Main reasons for accepting HPV vaccination

	Women accepting HPV vaccination (%)	
	
1. Reason	Age 16-20 years (N = 100)	Age 21-26 years (N = 101)
To prevent cervical cancer	84	83
Recommended by parents	33	13
Close relatives with cancer	18	14
Because it is available	21	10
Other^a^	5	19
Recommended by GP	6	11
High risk of cervical cancer	3	8
High risk of HPV infection	5	4
To prevent genital warts	5	4
Recommended by friends	2	4
Recommended by sibling	0	2
Recommended by partner	1	0

^a^Other included: various (e.g. work-related) experiences with cancer, vaccine paid for by parents or participating in a vaccine trial.

In the focus groups, personal experiences with HPV-related diseases could be a driver for accepting HPV vaccination. While the younger women knew little about cervical dysplasia and cervical cancer, the older women were better informed. They knew, for example, that cervical dysplasia does not necessarily progress to cancer, but that it can be highly distressing (see also [54]). Several of the older focus group participants knew that they had already been exposed to HPV: one woman had been diagnosed with HPV, two had had cervical dysplasia and one had an ex-partner who had genital warts.

Only few of all participants stated prevention of genital warts as a reason to be vaccinated against HPV (for the survey results see Table 1). The focus group participants explained that genital warts is a taboo disease that is given less public attention than other sexually transmitted diseases such as Chlamydia and AIDS (see also [55,56]), but when informed, they generally welcomed the fact that HPV vaccination can also prevent genital warts as an additional benefit.

#### The importance of significant others in decision-making

The survey showed that women who accepted HPV vaccination had discussed the vaccine with other people much more frequently than those who refused HPV vaccination: 92% compared with 70%. More women who accepted HPV vaccination had talked with their parents (the survey did not distinguish between fathers and mothers) and friends than those who refused HPV vaccination (Table 2). Also, more of the women accepting HPV vaccination had talked about the vaccine with their GP than those who refused it. The other people that the women talked with were typically other family members (Table 2).

**Table 2:** Who did you talk to about HPV vaccination?

	Women accepting HPV vaccination (%)		Women rejecting HPV vaccination (%)	
	
	Age 16-20 years (N = 100)	Age 21-26 years (N = 101)	Age 16-20 years (N = 102)	Age 21-26 years (N = 132)
Parents	72	68	42	27
Friends	64	60	50	47
General practitioner	16	34	5	8
Siblings	12	21	14	9
Partner	1	10	2	4
Others	3	4	4	5
Colleagues	0	4	1	5
School/college doctor	0	1	0	0

In the survey, many more women accepting HPV vaccination than women rejecting vaccination said to have heard about the vaccine from their parents. Another major difference between the groups was that the women rejecting HPV vaccination had mainly - and much more frequently than women accepting vaccination - heard of the vaccine on TV (Table 3).

**Table 3:** Where did you hear about HPV vaccination

	Women accepting HPV vaccination (%)		Women rejecting HPV vaccination (%)	
	
	Age 16-20 years (N = 100)	Age 21-26 years (N = 101)	Age 16-20 years (N = 102)	Age 21-26 years (N = 132)
Parents	50	31	13	8
TV	31	39	61	68
Friends	28	27	40	32
GP	21	28	19	18
Posters/brochures	14	15	14	22
Place of education	12	6	19	16
Other^a^	13	16	5	7
Internet	4	6	7	10
Siblings	4	5	8	2
Radio	1	3	2	7
Place of work	1	2	2	3
Partner	1			2

^a^Other mostly including other family members and fellow students

The survey showed that parents of women accepting HPV vaccination tended to have a higher level of education than parents of women refusing:

• *left school by the age of 16: *3% and 4% vs. 9% and 11%, for mothers and fathers respectively;

• *had a university degree*: 24% and 38% vs. 14% and 18%, for mothers and fathers respectively.

The focus group participants specified they had talked about the vaccine mainly to their mothers and female friends, but it was their mothers who were most influential, particularly for the younger participants, when they took the initiative to seek information and make an appointment with a doctor for their daughters. In contrast, almost half of the older focus group participants had spoken to their GP about HPV vaccination at their own initiative, and many expressed surprise that their GP had not talked to them about the vaccine and seemed to have poor knowledge of the subject when asked.

#### HPV vaccination seen as a fresh start

The focus group of women aged 21-26 years accepting HPV vaccination said that many of their peers were not being vaccinated because they thought it was already too late. Many of these participants, however, considered that with HPV vaccination and a negative cervical-smear result, they could make a fresh start:

You kind of have to say: well, this is where we start afresh. With all those people I meet from now on, I'm protected, right? I can't change what I've already done. [24-year-old focus group participant accepting HPV vaccination]

The younger focus group participants, in particular, said that they would actually like to know more about the benefits of the vaccine in sexually active women. They mostly understood that the more sexual partners a woman had had, the higher the risk that she had already been exposed to HPV and therefore the lower the expected benefit from vaccination. Although most of the 16-20-year-old focus group participants who had been HPV vaccinated were already sexually active, they hoped that they might still benefit from the vaccine.

I had already been with someone when I got the vaccine, so I was a bit like, shall I get it when there's a risk that it won't even do any good? But then I thought that I might as well get it while there's still a chance of preventing something. [16-year-old focus group participant accepting HPV vaccination]

A lot of my friends are also getting the vaccine because the attitude is like, if we wait for another 2 or 3 years, well, then we will probably get it [an HPV infection]. The chances that we're not with one, two, three partners within a couple of years, well, it's small, after all. So, we're right in that age group where, either, you get it now, or the chances that you get infected are simply too high. [20-year-old focus group participant accepting HPV vaccination]

#### Cost versus vaccine benefits for sexually active women

Most of the focus group participants accepting HPV vaccination said they had been vaccinated simply because the vaccine was available and they had no concerns about possible side-effects. Only the cost of the vaccine had made them hesitate. For almost all the younger and most of those older focus group participants, the vaccine had been partially or entirely paid for by their parents; many of the younger women said they would probably not have been vaccinated if they had had to pay themselves. The women in the focus groups explained that they had weighed up the benefits of vaccination after the onset of sexual activity against the cost and the balance tipped in favour of vaccination when parents helped out financially.

I also think it's got to do with the money, whether you'll spend it if there's a great risk that you've already been infected [with HPV]. My parents paid for it, so to me, it was like...well, I'd just like to get it. [16-year-old focus group participant accepting HPV vaccination]

I had already been with someone before I got the shot and I knew that it didn't protect 100%. But it was still an advantage to get it. And I think that the reason I got the shot was because my mother insisted that I was going to get it and that she'd pay. But I think that if I had to pay for it myself or if she hadn't been so keen that I should have it, I don't think anything would have come of it. Because I'm a bit sluggish some times. I often say to myself that I should get a bicycle helmet, and I've said so for years because I bike so much and it would be an obvious advantage to me. But I just never have got one. So, I think the same could have happened with this [24-year-old focus group participant accepting HPV vaccination]

### Results across the quantitative and qualitative studies: Barriers to HPV vaccination

In the survey, among women rejecting HPV vaccination, cost was cited as the greatest barrier to accepting HPV vaccination, particularly for the younger participants (Table 4). The second most frequent reason given was that it was too late, particularly for the older survey participants. Only a few survey participants said they were against vaccines in general, feared side-effects or found it too inconvenient to have three injections within six months. Very few women in the survey refused the vaccine because they thought they had a low risk of infection.

**Table 4:** Main reasons for rejecting HPV vaccination

Reason	Women rejecting vaccination (%)	
	
	16-20 years old (N = 102)	21-26 years old (N = 132)
Vaccine too expensive	72	51
Too late/already sexually active	15	41
Other^a^	20	26
Low risk of cervical cancer	9	6
Low risk of HPV infection	6	4
Fear of side-effects	3	7
Against having too many vaccines	5	3
Against all vaccinations	3	0
Don't have time for it	2	0
Inconvenience (three injections within six months)	2	0
Too young/not yet relevant	1	0

^a^Other: being already sexually active or not being in the target group.

#### Lack of information

Many of the focus group participants rejecting HPV vaccination explained that all the vaccines they knew were recommended as part of the childhood-immunisation programme or required for travel to certain destinations. Therefore, many of them found it strange that the decision to be HPV vaccinated was theirs.

I think it's strange...I've never had a vaccine that wasn't required or that I had to have because I was going to be travelling. Then suddenly a vaccine arrives that we can choose to have or not. And I think that's difficult because that's not something you just do. It's probably best to find out what it does. What is involved? Does it even protect me against this disease, or what? I think that's the odd part of this vaccine. It's a bit different from what we're used to. [17-year-old focus group participant refusing HPV vaccination]

These focus group participants felt they should be better informed before taking the decision because it was optional. They needed information like:

• Which diseases does it prevent?

• Does it work when you've already been sexually active?

• Is there any way of knowing whether you've been exposed to HPV?

• Why aren't boys vaccinated against HPV?

The focus group participants rejecting HPV vaccination generally wished that they had received the vaccine via the childhood-immunisation programme when it was free of charge and would certainly have been effective.

Most people don't know that much. They can't come to a decision. But I know a few people who got it as soon as they heard of it. Not many people I know are entirely against it. They hesitate because they don't know enough about it. [18-year-old focus group participant refusing HPV vaccination]

#### Not being in the target group

The focus group participants aged 21-26 years who refused HPV vaccination were under the impression that they were not in the target group. Most had not actively decided against being vaccinated or considered that they might already be infected with HPV; they simply thought that the vaccine was not targeted to women after sexual debut. Some said they thought that it was too late if they had had more than five sexual partners.

My choice isn't really an active one, so it's not like I don't want the vaccine as such. It's mostly that I thought that that ship had already sailed, that it was simply too late, so that the money would be wasted. [26-year-old focus group participant refusing HPV vaccination]

In addition, the women who were in steady relationships in the focus group of 21-26-year-olds thought that this excluded them from the target group; they particularly felt safe if they had also had a negative cervical-smear result. The fact that almost one-third of the survey participants refusing HPV vaccination had not discussed the vaccine with anyone, could be due to this consideration that the vaccine was not relevant to them.

#### Cost versus vaccine benefits for sexually active women

Many of the younger focus group participants said that if the vaccine was cheaper or free of charge they would have been vaccinated. The few women who were not yet sexually active said they hoped to be able to afford the vaccine before it was too late.

It's because you have to pay for it yourself. The reason that I'm in this group, clearly, it's not that I don't want this vaccine. Of course I do. But I can't afford to pay 3500 Danish kroner [470 euros] for it. Many people of our age, well, they're young people, still students, and perhaps they have a part-time job. But only few girls around the age of 16 can afford it, if they have to pay for it themselves. [20-year-old focus group participant refusing HPV vaccination]

The focus group participants rejecting HPV vaccination said they needed clear recommendations from the health authorities, their own GP or independent medical societies such as the Danish Cancer Society to justify spending so much money on the vaccine. None of their GPs had recommended that they should be vaccinated. Several of these focus group participants were sure that their parents would help to pay for the vaccine if they were convinced that it was beneficial. In other words, their decision was also determined by the balance between cost and the perception of benefit. The focus group participants rejecting vaccination stated that the absence of recommendations significantly decreased their willingness to pay, but if the vaccine was free of charge, they would feel less need for knowledge.

The cost stops many of my friends, but they also feel uncertain of whether they're in the target-group - whether you can even get it if you've been sexually active. To me, the price is decisive. If the vaccine was free, I would have just rushed to the doctor and had it done no matter if it worked or not - or how sexually active I'd been. I would have just got it over and done with. But because of the price, no matter what, I'm not going to spend 3500 Danish kroner [470 euros] on it, that's for sure. [18-year-old focus group participant rejecting HPV vaccination]

When asked, the other women in this focus group agreed that they would have been vaccinated if the vaccine was free of charge.

Some of the younger focus group participants rejecting vaccination reflected that not knowing any women of their age who had had cervical-cell changes or cervical cancer also made the threat of HPV infection more distant. Among the older focus group participants, many knew of women who had had cell changes but none had knowingly been exposed to HPV themselves. This suggests that a lack of personal experience with HPV infection can indeed act as a barrier to vaccination.

The focus group participants rejecting HPV vaccination did not themselves raise the subject of genital warts. When asked, only one of the younger and three of the older women had heard that the vaccine could prevent genital warts. Their knowledge about genital warts was generally lacking and their prevention was not part of the focus group participants' considerations regarding HPV vaccination.

#### The interpretation of health-policy decisions

The fact that, in Denmark, HPV vaccination is free of charge only for girls aged 12-15 years was, according to some focus group participants, a question of economic priority: the vaccine was offered to those who were sure to benefit because they had not yet become sexually active. However, many focus group participants were under the impression that the choice of target group was based on a purely medical assessment: the vaccine would have been free for older girls/women if there was a chance that this age group might benefit from it. By the same reasoning, some focus group participants assumed that the lack of information provided to women older than age 15 years was an indicator that the vaccine was not relevant in this case.

I think that only two of my friends have had the vaccine because...the fact that it's paid for up until the age of 15, I think, gives many of my friends aged 20-21 the impression that if you didn't get it then, it doesn't help. You're too old for it. That's kind of the signal that's sent by only giving it to girls up to age 15 years. A lot of my friends don't even consider it because they've got that impression. [20-year-old focus group participant rejecting HPV vaccination]

When it isn't free of charge for us, you assume that we're not in the group that it would help otherwise, it would surely have been free of charge for us as well, if it had any effect. When it's not, you think that it's like those people who get an influenza vaccine although they're not even at risk, that it's probably overkill to get this vaccine, because it's too late anyway. [24-year-old focus group participant rejecting HPV vaccination]

Yes, that's sort of the implicit message: that we don't get it for free because it doesn't help. That if it might help us, at least, we'd receive some sort of subsidy or we'd be recommended to go and get it. [another 24-year-old focus group participant rejecting HPV vaccination]

### The effect of vaccination on other methods of preventing HPV-related diseases

The focus groups pointed to the potential effects of HPV vaccination on other means of preventing HPV-related diseases. The women rejecting HPV vaccination believed that use of condoms was important to prevent all STIs, but not all were aware that condom use can only reduce and not entirely eliminate the risk of HPV infection. Moreover, many mentioned the fact that the use of condoms is often discarded after a few months in a steady relationship.

Many of the older focus group participants rejecting vaccination pointed to the cervical cancer-screening programme as a means to detect cell changes at an early stage and prevent cervical cancer. Some worried that HPV vaccination might produce a false sense of security that could cause some vaccinated women to abandon condom use or participation in the screening programme.

When asked, most focus group participants accepting HPV vaccination said that women should still have regular cervical-smear tests. However, their impression that the vaccine did not confer total protection was, again, related to the effectiveness of vaccination after the onset of sexual activity rather than to an understanding that the vaccine protects against only the two most common HPV types that cause cervical cancer and not against those causing the remaining 30% of cases. One focus group participant thought that the importance of screening would decrease because HPV vaccination would now prevent most cases of cervical cancer. Others thought that they might themselves become less concerned about attending check-ups, because of an increased sense of security after vaccination.

Perhaps, I'll become a bit more easy-going about it, like, now that I've got the vaccine, I'll feel a bit safer. [16-year-old focus group participant accepting HPV vaccination]

At the very least, I'm going to get my first check when I turn 23. I'm definitely going to do that just to make sure. I could have had the virus before I got the vaccine. But after that, I imagine that I'll be a bit more...well, I'll feel that I won't need it every three years, at least. I'll probably become a bit more careless [20-year-old focus group participant accepting HPV vaccination]

These statements went uncontested in the focus group and seemed to make sense to more of the younger participants. Although most focus group participants accepting HPV vaccination said that they would still use condoms to prevent other sexually transmitted diseases and pregnancy, those in steady relationships said they felt safer after vaccination, and this contributed to the sense of making a fresh start.

## Discussion

In this study of drivers and barriers to young women's choices about HPV vaccination, a literature review, a telephone interview-based survey and interviews with focus groups were combined methodologically. This triangulation of methods was used to ensure the validity and reliability of the study in the sense that it built upon existing knowledge, it sought to answer the research questions quantitatively by means of the survey and qualitatively to obtain an in-depth understanding of women's considerations.

The telephone survey provided quantitative data about the women accepting and rejecting HPV vaccination, including how many of those who wished to be vaccinated actually started the vaccination series and information about the most important factors influencing women's acceptance or rejection. The strength of focus groups lies in the insights they provide into the social forces acting on individual understandings of the area of interest. Interviews with focus groups in this study have produced a multi-faceted view of the ways in which Danish women's considerations about HPV vaccination relate to broader socio-cultural perceptions of cancer, sexuality and norms of equal access to health care. However, the social control that characterise group interviews mean that focus groups are less suitable for producing data about individual lives or narratives; for example, atypical individual understandings may be under-reported in focus groups [48].

This is one of the first studies to be conducted after the quadrivalent HPV vaccine became readily available in Denmark. It sheds light on the relationship between intention and action and the balance of factors that can encourage or hinder the translation of the intention to be vaccinated into the action of initiating vaccination in this specific socio-cultural context. While we believe that the many of these results are likely to be relevant and applicable to other societies, this thorough analysis of what is at stake in Denmark implies that they may not all be directly transferable to other countries. Perceptions of illnesses and preventive measures are always related to the specific socio-cultural context and health care system - and in this case also the particular information campaign. Whether the results of this study can be transferred to other societies thus remains to be shown.

This survey showed that most young Danish women have heard of vaccination against cervical cancer, but nearly one in five has not yet decided whether they want to be vaccinated. More than half of the women interviewed by telephone said they wanted to be vaccinated, but only half of these had actually started or completed the vaccination series. Since earlier studies found in the literature review have pointed to a very high acceptance rate, internationally and in Denmark, this suggests that the difference between intention and action can be considerable. Approximately one third of the women interviewed by telephone rejected HPV vaccination, but the focus groups suggested that this picture could change, provided that the barriers to vaccination were addressed.

This study did not show any opposition to the HPV vaccine as such, and participants in the qualitative study all had positive views on vaccination against cervical cancer. This also mirrors the high level of vaccine acceptance reported in earlier studies. Our participants mostly considered HPV vaccination as a means to prevent cervical cancer, unsurprisingly, since this has been the focus of the Danish information strategy. The focus groups revealed that the actual risk of acquiring cervical cancer was not considered. However, as suggested by other studies [21,24,40], our results show that women who have previously and knowingly been exposed to HPV-related diseases, or who know of people who have had HPV-related diseases or cancer, tend to be more accepting of vaccination.

Knowledge about genital warts was very limited among the focus group participants. This finding is supported by a recent study in which only 0.7% of Danish women assigned HPV as the cause of genital warts [57]. Thus this factor was not part of the women's consideration about HPV vaccination, although the fact that the vaccine could prevent 90% of cases of genital warts was seen as an additional benefit. Other studies have suggested that the information that the quadrivalent vaccine can prevent genital warts considerably increases men's acceptance of HPV vaccination [39], and our qualitative study indicates that the same effect might be observed for women.

Attitudes to the fact that HPV is transmitted sexually are highly dependent on the prevalent sexual morals in a society and need further investigation. In contrast to the results of some USA-based studies [11,13,16,19-21], for example, Danish men and women do not consider the mode of transmission of HPV as a barrier to HPV vaccination. On the contrary, they found that the mode of transmission increased the relevance of HPV vaccination of both sexes [10]. The present study also suggests that Danish women do not regard sexual abstinence before marriage as a realistic means of prevention of HPV infection.

The qualitative study confirmed earlier reports that Danish women's knowledge about HPV, cervical dysplasia and the properties of the HPV vaccine is generally poor. For example, in one study only 1.2% of Danish women were able to name HPV as the cause of cervical cancer [57]. Our study reveals that most focus group participants were under the impression that HPV vaccination provides full protection against cervical cancer, if given before the start of sexual activity. It is important that this misconception be investigated further since, although it may promote the uptake of vaccination, it may also encourage a false sense of security, e.g. some focus group participants indicated that they would be less concerned by the risk of HPV infection after HPV vaccination, especially if they had concurrently received a negative smear-test result. This highlights the danger that HPV vaccination might reduce uptake of other preventive measures, such as use of condoms and participation in the cervical cancer-screening programmes, which should ensure the maximal public health benefit for HPV vaccination.

Most of the focus group participants who had rejected HPV vaccination said they would actually like to be vaccinated if they could be convinced of the benefits. The most important factors influencing rejection were uncertainty or ignorance of the benefits of vaccination after the onset of sexual activity and the cost, which many regarded as very high. This was a confirmation of the results of the Danish HTA showing that young women were not likely not be vaccinated if they were to pay for it themselves. Our qualitative study shows that the lack of knowledge was less important when the vaccine was paid for by someone else, but became a decisive factor when the young woman had to pay herself.

We report that parental advice and financial support is very important to young women's acceptance of HPV vaccination. Proactive mothers, in particular, had great power to persuade their daughters to take active steps towards vaccination. The survey showed that acceptance of vaccination was associated with a higher level of education of the parents, although it is impossible to say whether the choice was influenced by the parents' educational level or by the potential higher income. GPs tended not to initiate talks with the young women about HPV vaccination, but when they did, or were consulted for this reason, they could also significantly influence the woman's choice. In contrast, women in the survey whose primary source of information was the media tended to think that HPV vaccination was not relevant to them, that they were insufficiently informed and consequently more frequently rejected vaccination.

The high coverage of the free HPV-vaccination programme for girls aged 12-15 years in Denmark supports the finding that the cost is very important to women's acceptance of vaccination. The focus group participants in our study had great confidence in the Danish health authorities and inclusion of HPV vaccination in this programme was regarded as approval of the safety and effectiveness of the vaccine. Conversely, they considered the decision not to include or inform women above the age of 15 years as an indication that this vaccine was not medically recommended for older girls or women. Our study shows that the fact that HPV vaccination is optional for women over 15 years old raises questions in it self and provokes uncertainty that can result in rejection, even when these women or their parents are able to afford vaccination. As other studies have shown, Danish women have a strong expectation that their GP will inform them of any necessary preventive health care measures, such as HPV vaccination [57]. In light of the relatively high cost of the vaccine, medical authorities and health-care professionals must disseminate clear recommendations and information about HPV vaccination targeted to young women who are already sexually active if such women, or their parents, are to be convinced of the benefits of vaccination and be willing to pay.

## Conclusions

Our survey among Danish women aged 16-26 years who have heard of HPV vaccination shows that nearly a fifth had not yet decided whether they want to be vaccinated and only a quarter had actually started or completed vaccination. As results from earlier studies reported that the intended acceptance was 70-90%, our results suggest that the difference between intention and action is considerable. Noteworthy drivers to acceptance of vaccination are parental financial support and recommendation by parents, medical authorities or a general practitioner. If the most important barriers to HPV vaccination - namely, the cost and the lack of information about the benefits of vaccination for sexually active women - were to be addressed, it is likely that uptake of HPV vaccination would increase significantly in Denmark. This would accelerate the reductions in incidence of cervical dysplasia, cervical cancer and genital warts already expected to be achieved by including the HPV vaccine in the childhood-immunisation programme.

## Competing interests

GLM has received a research grant for this study from Sanofi Pasteur MSD.

## Authors' contributions

GLM conceived the study and its design, carried out the data collection, analysed and interpreted the data and drafted the manuscript.

## Author information

GLM has a MA in social anthropology and is specialised in medical anthropology. She currently works as a research consultant at AnthroConsult specialised in qualitative patient studies.

## Pre-publication history

The pre-publication history for this paper can be accessed here:

http://www.biomedcentral.com/1471-2458/10/68/prepub
